# Modeling the intention and donation of second-hand clothing in the context of an emerging economy

**DOI:** 10.1038/s41598-023-42437-y

**Published:** 2023-09-13

**Authors:** Mengling Wu, Abdullah Al Mamun, Qing Yang, Jingzu Gao, Muhammad Khalilur Rahman, Sayed Samer Ali Al Shami

**Affiliations:** 1 UKM-Graduate School of Business, Universiti Kebangsaan Malaysia, 43600 Bangi, Selangor Darul Ehsan Malaysia; 2 Faculty of Entrepreneurship and Business, Universiti Malaysia Kelantan, Pengkalan Chepa, Malaysia; 3 Angkasa-UMK Research Academy (AURA), Universiti Malaysia Kelantan, Pengkalan Chepa, Malaysia; 4 Institute of Technology Management and Entrepreneurship, Universiti Teknikal Malaysia Melaka, Melaka, Malaysia

**Keywords:** Human behaviour, Sustainability

## Abstract

The culture of fast fashion accelerates the consumption rate of individuals but at the expense of significant environmental stress. With a large amount of discarded clothing accumulating in landfills, it is crucial to encourage people to dispose of second-hand clothing (SHC) as sustainably as possible, especially in an emerging economy with large volume consumption. Through a survey of 619 respondents from China, this study explored the factors affecting people’s intentions and actual donation behaviors toward SHC. It extends the theory of interpersonal behavior (TIB) with environmental factors to construct a research framework, which included cognitive factors (attitude towards sustainable consumption), social factors (sense of community) and personal factors (perceived hedonic benefit) under TIB and the environment factors refers to problem awareness and ascription responsibility. Partial least squares structural equation modeling was employed to analyze the data. The findings revealed that attitudes toward sustainable consumption, problem awareness, ascription of responsibility, sense of community, and perceived hedonic benefit significantly and positively influenced people’s intentions and practices of SHC donation. This study will aid governments and relevant green environmental protection organizations in formulating more precise strategies for sustainable development, and promote relevant research on the sustainable disposal of SHC.

## Introduction

In the coming decades, climate change and environmental pollution will become formidable obstacles for humanity. Excessive energy consumption is considered to be the primary factor hindering sustainable development and enabling this trajectory^1, 2^. The rapid change in fashion and people’s pursuit of it has created a one-time trend in the clothing industry. The promotion of technology and interests has caused clothing enterprises to produce quickly at maximum capacity, resulting in a market cycle that lasts only a few weeks. Low-priced clothing has a difficult time gaining consumers’ long-term favor, as they frequently discard it without a second thought. This trend of fast fashion has contributed to global overconsumption and exacerbated the prevalence of a disposable consumption culture, which is regarded as the primary cause of the increase in global clothing disposal volume^3, 4^. According to some studies, the fashion industry is responsible for 10% of the world’s carbon dioxide emissions and close to 20% of the world’s wastewater emissions. With the continued development of the textile industry in Asian and African markets, these numbers will continue to rise, which will undoubtedly increase the ecological burden and result in further environmental degradation^5^.

Product disposal is the process by which consumers decide to stop using items that still have value. They abandon the use of the product through various means, such as storing, selling, discarding, gifting, or donating^6^. The way of SHC is disposal determines whether it is reused or sent to garbage dumps or landfills. Certain synthetic fibers possess distinct material properties that pose challenges in terms of reusability and ecosystem degradation, even though natural fibers used as raw materials for sustainable fabrics decompose, they still contribute to the emission of greenhouse gases. Nevertheless, the predominant method of disposing of SHC in most countries continues to be landfilling^5^. Despite ongoing efforts by society and government to encourage enterprises to adopt sustainable raw materials and production methods, recent studies have indicated that addressing the sustainability issue within the textile and clothing industry should extend beyond the realms of production and consuming. It should also encompass the sustainable disposal of clothing^7, 8^. Sustainable SHC disposal involves maximizing the length of the clothing life cycle and minimizing the amount of SHC disposed of in landfills or incinerators. For residents, it refers to the transfer of SHC through means such as donating to charity or secondhand stores, renting, inheriting, trading, swapping, or borrowing^4^. In their comprehensive literature review on waste treatment in the textile industry, Shirvanimoghaddam et al.^7^ emphasized the significance of incorporating recycling and reusing strategies for used textiles. They argued that such practices offer an ideal approach for resource recovery, restoration, and the maximization of product value.

China is the world's largest textile producer, consumer, and exporter, producing nearly 100 million tonnes of textile waste per year ^9^. The massive population has also resulted in a boom in China's local clothing industry, as well as an abundance of second-hand clothing. Under the pressure of the carbon emission limit, China is currently developing policies on second-hand clothing donation and recycling. As a sustainable way to dispose of secondhand clothing, the donation of SHC has a unique value in promoting social welfare, reducing resource consumption, and minimizing environmental impact. Traditional SHC donation methods include government and non-profit clothing recycling programs, some organizations collecting their own brands of clothing, and second-hand trading stores selling clothing^6^. With the advancement of Internet platforms and the logistics industry, some online platforms have emerged as new channels for SHC donations. After users make an appointment to donate SHC, some platforms offer free door-to-door services, making the process of donating clothing more convenient and quicker. This broadens the options for SHC donation in China and increases the convenience of SHC donation^6^. Although the government and businesses provide a good environment for the recycling of SHC, consumers, as the producers of SHC, play an irreplaceable role in improving the recycling rate of used clothing, as their disposal decisions determine the flow of used clothing^8^. However, when presented with multiple disposal options for secondhand clothing, Chinese consumers are more likely to discard or leave it at home. According to Zhang et al.^6^, in a survey of the manners in which Chinese residents dispose of secondhand clothing, it was discovered that nearly forty percent of respondents kept their clothes at home and discarded them in an unsustainable manner. In the long term, this phenomenon represents a significant depletion of resources.

Most previous research on the sustainability of the apparel industry has focused on the purchase use or acquisition stage of apparel, whereas the impact of the unsustainable SHC disposal stage remains underappreciated^8, 10, 11^. Recycling as much unwanted SHC as possible remains a pressing issue in China. The current body of literature lacks comprehensive research on the sustainable disposal of SHC within the Chinese context, particularly in relation to donation practices. Lang and Zhang^12^ conducted a study that examined and compared the motivations of Chinese consumers in engaging in SHC acquisition, specifically in the activities of swapping SHC and swapping SHC with family and friends. Zhang et al.^6^ examined the behavioural intention of Chinese consumers in relation to the disposal of second-hand clothing via online platforms, in their study, they observed that the predominant practices of discarding and storing continue to dominant, and emphasized that China still possesses significant potential for the advancement of sustainable clothing disposal practices. Zhang and Dong^13^ investigated the impact of virtual social capital on sustainable SHC consumption patterns and highlighted the importance of social factors in shaping Chinese consumers' sustainable SHC consumption patterns in the context of the social media development. Based on the extant literature, it is evident that there remains a dearth of research investigating the determinants of residents' behaviour pertaining to second-hand clothing donation in China, an emerging economy. It is noteworthy that the study of pro-environmental behaviour frequently employs psychologically grounded theories, such as the Theory of Reasoned Action (TRA) and the Theory of Planned Behavior (TPB)^14^. From an individual rationality standpoint, these theories place significant emphasis on the impact of attitude, norms, and other related factors on behavioral intention, and dem onstrating its effectiveness by numerous studies^14, 15^. However, these theories tend to overlook the role of social factors and irrational personal emotional factors in shaping behaviour. Consequently, the present study incorporates the Interpersonal Behavior Theory (TIB) as a supplementary framework. Furthermore, an oversight in the existing body of research utilising the TIB framework to investigate the domain of sustainability is the limited consideration given to the influence of environmental factors^16^^.^ Hence, this study incorporates environmental psychological factors (problem awareness, ascription of responsibility) into the research framework in order to enhance the comprehensiveness of predicting the factors that impact consumer donation to SHC.

To alleviate the impact of textile waste on the environment and partially fill the gap in existing research, this study aimed to explore the factors influencing SHC donation intention and the actual donation of SHC. To achieve this objective, this study proposed a model based on a combination of the TIB model and environmental psychology factors, including cognitive factors (attitude toward sustainable consumption, environmental factors (problem awareness and ascription of responsibility), social factors (sense of community), and personal factors (perceived hedonic benefit). This construct model was empirically tested in China. The remainder of this paper includes a literature review in the next section, an introduction of the research methodology adopted in this study in the third section, an interpretation and data analysis of the results in the fourth section, and finally, a discussion of the implications and conclusions in the last section.

## Literature review

### Theoretical foundation

In the TIB model, Triandis^17^ pointed out that an individual’s behavioral intention is mainly influenced by cognitive, social, and personal factors. Among these, attitude is an influencing factor of behavioral intention, which has attracted much attention in previous studies^18^. As mentioned by Ajzen^19^, attitudes show people’s views or perceptions of certain things and promote the generation of individual behaviors. Simultaneously, social factors are also emphasized in the TIB model. As social individuals, it is difficult for humans to escape the influence of society; therefore, social factors are key in the study of pro-environmental behavior. In addition, when previous popular models studied individual behavioral intentions and related behaviors from a psychological perspective, most were based on decisions made by individuals under rational thinking, and few discussed the impact of individual emotions on behavioral intentions under irrational behavior^18^. Previous studies have confirmed that TIB has a strong ability to explain intentions and behaviors^16, 20^. Therefore, it is appropriate to apply TIB to the pro-environmental context of SHC donation, which considers rational attitudes, irrational emotions, and social impact. Based on the above theoretical background, although the TIB model has been applied to pro-environmental behaviors in some studies, it has not received much attention in combination with environmental psychological factors. Thus, this study included environmental factors based on the TIB model, making the research framework more adaptable to the pro-environmental context that refers to the sustainable disposal of SHC.

### Hypotheses development

#### Attitude toward sustainable consumption (ASC) and SHC donation intention (SDI)

Attitude refers to the degree to which a person evaluates a behavior positively or negatively^19^. It is the key component of various research models such as TPB and TIB^17, 19^. Positive attitude produces positive results, and vice versa. It is always significant when an individual’s sustainable behavior produces a positive and beneficial influence. Liu et al.^21^ verified that the status of consumers environment attitudes positively affects their environment behavioral intention. Research on second-hand product buying also confirms that attitudes toward sustainable consumption can increase consumers’ intentions to buy SHC^22^ or donate SHC^6^. Therefore, this study proposed the following hypothesis:

H_1_: ASC positively influences SDI.

#### Problem awareness (PRA) and SDI

PRA is the perception about the severity of the problems that individuals may meet during their life^23^. It is further defined as an individual’s awareness of predictable environmental problems. When people are aware of problems in the environment, they may consciously avoid these problems or participate more actively in solving them. In many existing studies on pro-environment files, PRA is considered the decisive factor in pro-environment behavioral intention or behavior^24, 25^. The rise of pro-environmental and pro-social movements has led to increased awareness of the detrimental effects of resource waste through social media platforms. Consequently, individuals are motivated to take action in order to mitigate the adverse consequences on the environment^23^. A similar perspective is also shared by Song et al.^25^, who argue that customers exhibit a greater intention towards reusing express packages when they become aware of the environmental issues stemming from inadequate handling practices. Accordingly, the following hypothesis is proposed:

H_2_: PRA positively influences SDI.

#### Ascription of responsibility (ASR) and SDI

The concept of ASR has been a critical topic in various studies related to prosocial behavior for a few years^25, 26^. ASR for this study is described as a sense of responsibility toward the negative impact of failing to engage in pro-environmental behaviors^26^. People determine what they should or should not do based on how much responsibility they are assigned. Consequently, ASR will help people become more inclined to adopt pro-environmental behaviors and, to some extent, deter them from engaging in environmentally destructive behavior. When Onwezen et al.^27^ studied pro-environmental behaviors, they emphasized the positive effect of responsibility-related factors on pro-environmental behaviors. Vaske et al.^28^ also proposed that farmers with a strong ASR have a greater intention to engage in conventional behaviors. Following the empirical studies, it is expected that once individuals ascribe to the responsibility to act to reduce environmental threats, they have more intention to donate SHC. Thus, this study proposed the following hypothesis:

H_3_: ASR positively influences SDI.

#### Sense of community (SOC) and SDI

SOC is an individual’s feeling of belongingness, which refers to the importance of both individuals and groups to each other, and that sharing of faith among individuals and collectives can be achieved through mutual effort^29^. Some researchers have defined SOC as the emotional bond that people have with society^30–32^. Overall, society is the ultimate beneficiary of pro-environmental behavior; therefore, it is essential to link the intention of sustainable development behavior with a SOC. Individuals who possess a robust emotional attachment to society are more inclined to exhibit psychological awareness and actively participate in environmentally sustainable behaviors^33, 34^. When examining the factors that influence people's participation in pro environment behaviors, researchers such as Schanes and Stagl^35^ discovered that identity and SOC were motivational factors in individuals’ intention to share food. Therefore, this study proposed the following hypothesis:

H_4_: SOC positively influences SDI.

#### Perceived hedonic benefit (PHB) and SDI

PHB refers to the happiness of an individual stemming from their behavior; it is a positive emotional outcome for customers^36^. PHB is an important motivation factor in an individual’s behavioral intention as it is the intrinsic emotional benefits^37^. Numerous empirical investigations have substantiated the notion that individuals are more likely to exhibit pro-environmental behaviours when they derive additional gratification from engaging in such activities^38–40^. After investigating the sustainable clothing disposal behavior of Indonesian consumers, Fenitra et al.^40^ also confirmed that emotional factors positively impact donation behavior. Therefore, a high PHB toward activities always indicates a high intention to conduct them. Hence, we proposed the following hypothesis:

H_5_: PHB positively influences SDI.

#### SDI and SHC donation behavior (SDB)

Intention is a stimulus or perceived subjective that originates from an individual and is typically regarded as the cause of behavior^21, 41^. Numerous theories cite intention as a factor that directly influences actual behavior, include the TIB, TPB, and technology acceptance model^17, 19, 42^. Usually, for a free and independent individual, behavioral intention frequently determines his final action. Previous studies on pro-environmental backgrounds have also confirmed the strong or at least moderate relationship between intention and behavior^21, 43–45^. According to a study conducted by Liu et al.^21^ about pro-environment behaviour, it was observed that Chinese residents exhibit a higher likelihood of translating their pro-environmental intention into actual actions. Therefore, this study proposed the following hypothesis:

H_6_: SDI positively influences SDB.

#### Mediating effect of SDI

Based on the TIB model and environmental psychological factors, this study proposed ASC, PRA, ASR, SOC, and PHB as important predictors of SDI based on the previous studies ^25, 28, 35, 40^. In addition, a relationship between SDI and SDB has also been founded by many researchers^21, 44^. Occasionally, an enduring disparity can be observed between an individual's psychological motivations and their manifested actions, and generating actual behavior is difficult if there is insufficient willpower. Previous research on green and sustainable consumption has also found that the degree of individual intention influences the validity of relevant predictors^21^. Therefore, this study predicts that SDI mediates the association between ASC, PRA, ASR, SOC, PHB, and SDB. The following hypothesis is proposed:

H_7a-e_: SDI mediates the relationship between ASC, PRA, ASR, SOC and PHB on SDB.

**Figure 1:**
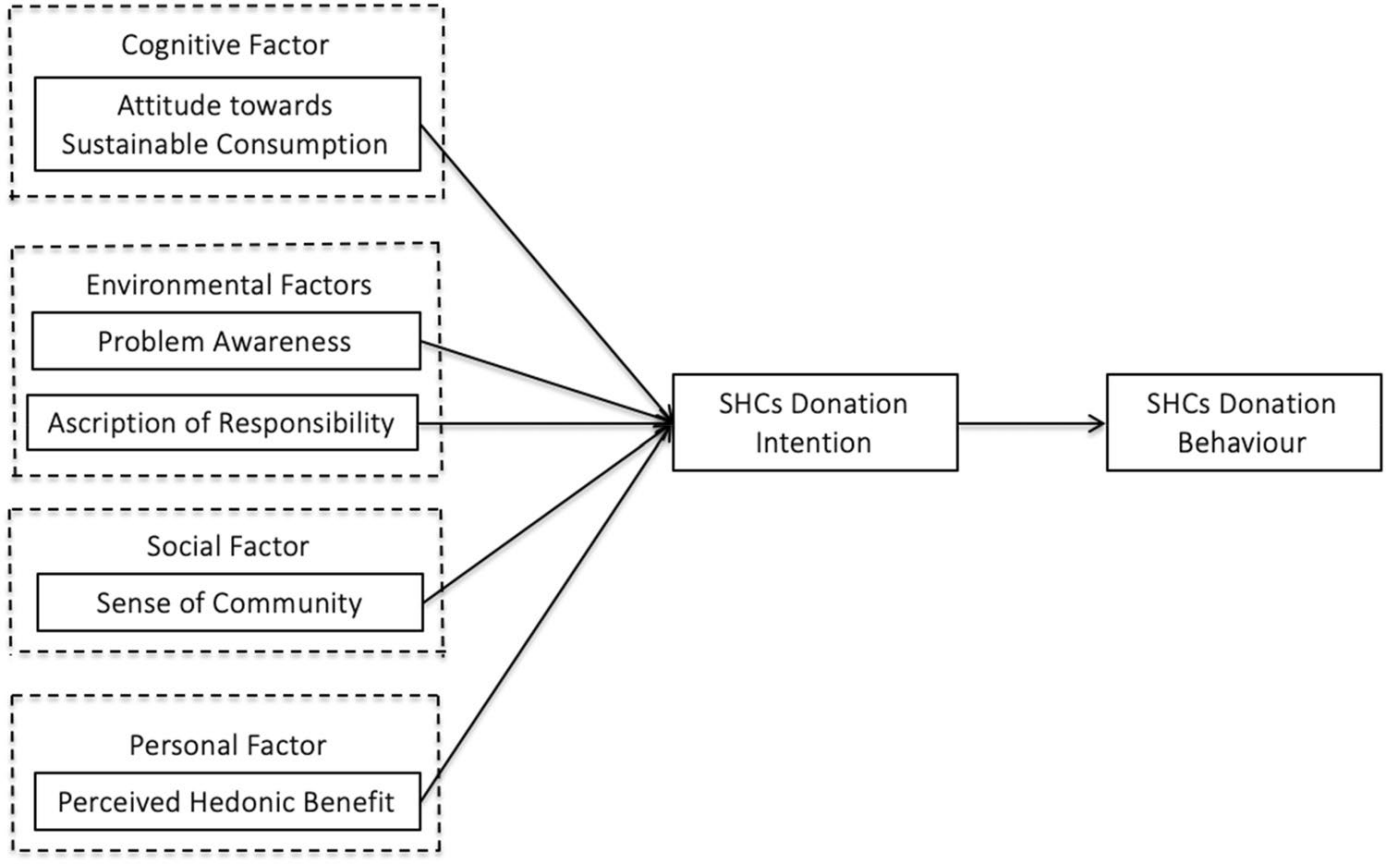
Research framework.

All associations hypothesized above are presented in Fig. 1 below:

## Research methodology

### Data collection

This study is a cross sectional research which invested the intention and behavior toward SHC donation among Chinese residence. Therefore, the target population was the residents in China. Based on supported by G*Power 3.1 with the 0.15 parameters of effect size, power of 0.95, and six predictors, the minimum sample size of this study was 146^46^. Convenience sampling was used to reach the target population as effectively as possible. The advancement of Internet technology has facilitated convenient and cost-effective access to a wide range of research participants. Additionally, certain studies have demonstrated that Internet samples exhibit greater representativeness in relation to demographic attributes^47^. Consequently, the present study employs an online survey as a means of data collection, spanning the period from June to July 2022. The questionnaire was uploaded onto the well-known online survey platform WJX (https://www.wjx.cn) in order to generate a survey link. Subsequently, the survey link was distributed randomly through a widely utilised social media platform in China, such as WeChat, QQ, or Micro-blog. Upon accessing the provided hyperlink, individuals will be prompted to fill up the informed consent, thereby confirming their comprehension of the study's objectives and significance, as well as the rights afforded to participants. By voluntarily agreeing to participate, individuals will proceed to the subsequent phase of completing the questionnaire. The remaining 619 valid responses were used for data analysis after the incomplete and unreasonable responses (respondents who provided the same response for all items) were eliminated.

### Survey instrument

The measurement items of all constructs were adapted from the previous literature to match the context of this study and ensure the validity and reliability of the measurement. The questionnaire was divided into two sections: the first section included demographic information such as gender, age, and education level; the second part comprised items related to each variable, measured using a seven-point Likert scale. The items used to assess ASC were adapted from Joshi et al.^15^ and Smith et al^48^. Items for ASR were adapted from Zhang et al.^49^ and Wang et al.^50^. The measurement tool for SOC was adapted from Li-Chun Hsu^51^ and Huang et al.^52^. The items for PHB were adapted from Hsu and Lin^53^ and Wiedmann et al.^54^. Both the measurement of PRA and SDB were taken from Khan et al.^55^. The items on SDI were adapted from Kim et al.^11^ and Putrevu and Lord^56^. All corresponding details are presented in the supplementary materials (S1. Research Instrument).

### Common method bias (CMB)

As a critical concern, Common Method Bias (CMB) could potentially jeopardize the reliability of research findings. The study employed ex-ante and post-hoc based on the recommendation of previous studies^57, 58^. During the data collection progress, the respondents were consulted using the concealed identity approach, which mainly involved the following steps: (a) Before the respondents engaged in the questionnaire survey, they were informed about the purpose of data collection, the ultimate use of the data, the non-commercial nature of the study, and the confidentiality of the questionnaire. (b) The respondents were informed of their right to withdraw from participating in the data collection at any time. (c) Anonymous questionnaires were used, and the respondents were not required to provide their names or contact information. (d) Considering that the research topic was related to environmental protection, to avoid respondents consciously selecting socially desirable "correct answers", they were informed at the beginning of the questionnaire that all options in the questionnaire had no distinction between correct and incorrect and were asked to provide responses based on their genuine thoughts. Finally, after obtaining informed consent, the study provided an explanation of the term "second-hand clothing" to the respondents, offering a detailed definition and explanation of it. The purpose of this module was to enable the respondents to understand the research context and, at the same time, alleviate any nervousness and apprehension they might have had before proceeding with the questionnaire.

Although thorough preventive measures were taken, the study employed post hoc remedies to assess the potential impact of remaining CMB on the final data results. Podsakoff and Organ^59^ proposed the Harman single-factor test as a common and reasonable post hoc remedy for CMB. In this study, the Harman single-factor test result showed that the variance explained by the first factor was 0.491, which did not exceed the recommended threshold of 0.50. Additionally, previous research acknowledges that the number of constructed items increases the values obtained from factor analysis. Therefore, this study considered more than one CMB testing method and used variance inflation factor (VIF) for collinearity testing, which is the most common approach in Partial Least Squares Structural Equation Modeling (PLS-SEM)^60^. The collinearity test finding presented in Table 1 showed that the maximum VIF value was 3.154, and below the recommended threshold of 3.3 according to Kock^60^. Considering the results of the Harman single-factor test and the full collinearity test, this study concludes that CMB is not a significant issue.

**Table 1:** Full collinearity test.

Variables	ASC	PRA	ASR	SOC	PHB	SDI	SDB
VIF	2.532	2.981	2.125	2.295	1.566	3.154	1.733

Source: Author’s data analysis.

*ASC* attitude toward sustainable consumption, *PRA* problem awareness, *ASR* ascription of responsibility, *SOC* sense of community, *PHB* perceived hedonic benefit, *SDI* SHC donation intention, *SDB* SHC donation behavior.

### Multivariate normality

As it is commonly used to test the potential non-normality of multivariate data, Mardia’s multivariate skewness kurtosis, recommended by Cain et al.^61^, was used in this study to test multivariate normality. (Source: https://webpower.psychstat.org/wiki/tools/index). The results of Mardia’s multivariate skewness and kurtosis show that the p-values of both skewness and kurtosis are less than 0.05, which indicates that the data are not normally distributed. Compared with other parametric analysis software, SmartPLS is more suitable for this study because it uses a non-parametric analysis approach^62^.

### Data analysis method

This study applied PLS-SEM for statistical data analysis, which uses separate ordinary least squares regressions to calculate partial regression relationships in the measurement and structural models^63^. It has appealed to many studies owing to its strengths, such as dealing with complex models that have many constructs or variables and being more robust with non-normality data analysis than with other parametrical approaches^62^. In order to account for detecting potential heterogeneity and testing the existence of differences across group-specific parameter estimate that could help researchers to identify meaningful differences, a multi-group analysis (MGA) was also performed, and this approach is also considered an effective method for evaluating moderation across multiple relationships^64^. Before conduction MGA, a permutation test that to assess measurement invariance through MICOM procedure was employed in this study. This approach was also recommended by Hair et al.^62^ as it is a non-parametric method and has the ability to control type I errors.

### Ethics approval

The human research ethics committee of Changzhi University approved this study (Ref. CZ-2022-080). This study has been performed in accordance with the Declaration of Helsinki.

### Informed consent to participate

Written informed consent for participation was obtained from respondents who participated in the survey. No data was collected from anyone under 18 years old.

## Findings

### Demographic characteristics

The sociodemographic characteristics of all respondents, including gender, age, education level, marital status, occupation, monthly income, buying clothing preference, and experience of SHC donation, are presented in Table 2. Females accounted for the largest number of participants, which was 344 (55.6%). Regarding age group, the largest group is the 20 to 30 age group, and there are 185 (29.9%) respondents who belong to it. The majority of respondents had a bachelor’s degree, with a total of 244 (39.4%). More than half of the participants were married, and the number of them is 384 (56.2%). Participants from eastern China accounted for the largest proportion, which is 115 (18.6%). For occupation, 195 (31.5%) respondents were private employees, and the number in this category is higher than others. For monthly income, the majority of participants receive an income ranging from 4501 to 6000 RMB, with a number of 137 (22.1%). 273 (44.1%) participants bought clothing one to two times a month, while 194 (31.3%) participants spent 200 to RMB400 on their clothing each month, and both of them belonged to the largest group. The vast majority of respondents had experience with SHC donation, with a total of 569 (91.9%).

**Table 2:** Profile of the respondents.

	N	%		N	%
Gender			Occupation		
Male	275	44.4	Unemployed	18	2.9
Female	344	55.6	Self-employed	148	23.9
Total	619	100	Student	108	17.4
			Housewife	54	8.7
Age			Privately employed	195	31.5
18–20	101	16.3	Public servant	96	15.5
20–30	185	29.9	Total	619	100
31–40	146	23.6			
41–50	130	21	Monthly income		
Above 50	57	9.2	Less than RMB1500	54	8.7
Total	619	100	RMB1500–RMB3000	73	11.8
			RMB3001–RMB4500	142	22.9
Education level			RMB4501–RMB6000	137	22.1
Diploma/advanced diploma	157	25.4	RMB6001–RMB7500	123	19.9
Bachelor	244	39.4	More than RMB7500	90	14.5
Postgraduate degree	107	17.3	Total	619	100
Others	111	17.9	Frequency of buying clothing		
Total	619	100			
Marital status			Less than 1 time	158	25.5
			1–2 times	273	44.1
Single	180	29.1	3–4 times	126	20.4
Married	348	56.2	More than four times	62	10
Divorced	67	10.8	Total	619	100
Widow	24	3.9	Monthly spending of clothing		
Total	619	100			
Location			Less than RMB200	110	17.8
			RMB200–RMB400	194	31.3
Northeast China	64	10.3	RMB401–RMB600	98	15.8
North China	64	10.3	RMB601–RMB800	106	17.1
East China	115	18.6	RMB801–RMB1000	63	10.2
Central China	105	17	More than RMB1000	48	7.8
South China	107	17.3	Total	619	100
Northwest China	79	12.8	Ever donated SHC		
Southwest China	67	10.8			
Others	18	2.9	Yes	569	91.9
Total	619	100	No	50	8.1
			Total	619	100

### Validity and reliability

The first step in evaluating the PLS-SEM results was to examine the measurement models. Cronbach’s alpha, composite reliability, and reliability coefficient ρ_A_(rho_a) have been widely recommended as the proper indication to evaluate internal consistency reliability^62^. According to the analysis results in Table 3, Cronbach’s alpha, composite reliability, and reliability coefficient ρ_A_ of all the constructs are above 0.7, indicating an acceptable level of internal consistency reliability. Moreover, the evaluation of convergent validity is also critical to ensure that the indicators of the reflective construct converge to a high proportion of variance, and the average variance extracted (AVE) is the traditional indicator to measure it^62^. As presented in Table 3, the AVE values of all seven constructs were higher than the threshold of 0.5, confirming the convergent validity of the measurement model.

**Table 3:** Reliability and validity.

	Items	Cronbach’s alpha	Composite reliability (rho_a)	Composite reliability	AVE	VIF
ASC	4	0.907	0.908	0.935	0.781	1.950
PRA	4	0.881	0.884	0.918	0.737	2.741
ASR	4	0.906	0.912	0.934	0.780	2.104
SOC	4	0.866	0.871	0.909	0.714	2.089
PHB	4	0.918	0.943	0.942	0.802	1.536
SDI	4	0.942	0.944	0.958	0.851	1.000
SDB	4	0.936	0.951	0.954	0.839	

Source: Author’s data analysis.

*ASC* attitude toward sustainable consumption, *PRA* problem awareness, *ASR* ascription of responsibility, *SOC* sense of community, *PHB* perceived hedonic benefit, *SDI* SHC donation intention, *SDB* SHC donation behavior, *AVE* average variance extracted, *VIF* variance inflation factor.

Discriminant validity measures the level of one construct that is distinct from others. As a traditional method, the Fornell–Larcker criterion can be used to evaluate discriminant validity, in which the square root of the AVE should be larger than the latent variable correlations^62^. Based on the results in Table 4, each square root of the AVE was higher than the correlations of the other constructs. Cross-loading was also employed in this study to assess discrimination validity, and the results are shown in Table 5. All the outer loadings were above 0.5, which is higher than other cross-loadings. The heterotrait-monotrait ratio (HTMT) is another accurate indicator for measuring discriminant validity; all HTMT values should be below the threshold of 0.9 to ensure discriminant validity^65^. According to Table 4, none of the HTMTs exceeded 0.9. Concluding all three measurement indicators, the discriminant validity of this study was confirmed.

**Table 4:** Discriminant validity.

	ASC	PRA	ASR	SOC	PHB	SDI	SDB
Fornell–Larcker criterion							
ASC	0.884						
PRA	0.644	0.859					
ASR	0.514	0.698	0.883				
SOC	0.566	0.637	0.561	0.845			
PHB	0.477	0.393	0.430	0.535	0.895		
SDI	0.724	0.720	0.610	0.678	0.505	0.923	
SDB	0.594	0.530	0.453	0.531	0.441	0.529	0.916
Heterotrait-monotrait ratio (HTMT)							
ASC							
PRA	0.719						
ASR	0.564	0.776					
SOC	0.635	0.726	0.628				
PHB	0.516	0.428	0.468	0.591			
SDI	0.780	0.788	0.655	0.748	0.532		
SDB	0.639	0.575	0.483	0.581	0.462	0.555	

Source: Author’s data analysis.

*ASC* attitude toward sustainable consumption, *PRA* problem awareness, *ASR* ascription of responsibility, *SOC* sense of community, *PHB* perceived hedonic benefit, *SDI* SHC donation intention, *SDB* SHC donation behavior.

**Table 5:** Loading and cross loadings.

	ASC	PRA	ASR	SOC	PHB	SDI	SDB
ASC1	0.879	0.584	0.497	0.560	0.489	0.656	0.553
ASC2	0.872	0.529	0.422	0.446	0.370	0.591	0.493
ASC3	0.887	0.570	0.434	0.453	0.391	0.641	0.504
ASC4	0.897	0.590	0.460	0.534	0.432	0.667	0.548
PRA1	0.580	0.880	0.681	0.621	0.452	0.674	0.512
PRA2	0.517	0.865	0.560	0.508	0.323	0.604	0.412
PRA3	0.555	0.852	0.569	0.501	0.318	0.590	0.433
PRA4	0.558	0.838	0.580	0.549	0.244	0.598	0.458
ASR1	0.468	0.630	0.885	0.572	0.378	0.587	0.427
ASR2	0.437	0.588	0.869	0.501	0.359	0.495	0.384
ASR3	0.494	0.685	0.923	0.507	0.402	0.570	0.426
ASR4	0.409	0.555	0.854	0.387	0.378	0.490	0.359
SOC1	0.528	0.583	0.520	0.891	0.480	0.633	0.476
SOC2	0.537	0.509	0.413	0.819	0.448	0.553	0.409
SOC3	0.446	0.527	0.499	0.825	0.450	0.541	0.452
SOC4	0.396	0.530	0.459	0.843	0.429	0.558	0.455
PHB1	0.494	0.424	0.448	0.578	0.944	0.558	0.470
PHB2	0.397	0.364	0.420	0.414	0.877	0.390	0.336
PHB3	0.416	0.326	0.372	0.456	0.880	0.409	0.382
PHB4	0.385	0.278	0.288	0.439	0.879	0.419	0.368
SDI1	0.710	0.680	0.594	0.656	0.509	0.935	0.544
SDI2	0.595	0.622	0.508	0.586	0.422	0.905	0.467
SDI3	0.687	0.671	0.564	0.630	0.449	0.926	0.497
SDI4	0.672	0.680	0.579	0.626	0.480	0.924	0.438
SDB1	0.598	0.554	0.484	0.578	0.473	0.559	0.940
SDB2	0.550	0.476	0.411	0.452	0.422	0.481	0.926
SDB3	0.554	0.500	0.422	0.506	0.394	0.483	0.934
SDB4	0.458	0.392	0.321	0.379	0.301	0.391	0.862

Source: Author’s data analysis.

*ASC* attitude toward sustainable consumption, *PRA* problem awareness, *ASR* ascription of responsibility, *SOC* sense of community, *PHB* perceived hedonic benefit, *SDI* SHC donation intention, *SDB* SHC donation behavior.

### Hypothesis testing

Before testing the hypotheses using path coefficient, a collinearity test must be performed to ensure that the construct model is free from substantial effects, and the VIF value of each construct must be below 5^62^. According to Table 3, the VIF value range of all constructs is 1.536–2.741, which is lower than 5, indicating no collinearity issue in this study. The path coefficient indicates the importance of each predecessor construct to the target construct. As presented in Table 6, the standardized path coefficients and significant level of ASC (β = 0.345, p < 0.01), PRA (β = 0.216, p < 0.01), ASR (β = 0.091, p < 0.05), SOC (β = 0.223, p < 0.01), PHB (β = 0.079, p < 0.05) have indicated that all five variables have a significant positive influence on SDI, and the confidence interval (CI) of each hypothesis did not contain zero between level 5% and 95%. Therefore, H1, H2, H3, H4, and H5 were supported. Furthermore, the SDI was found to positively and significantly affect SDB (β = 0.529, p < 0.01), thus supporting H6.

**Table 6:** Hypothesis testing.

	Hypothesis	Beta	t	p	Confidence intervals	R^2^	f^2^	Decision
Factors affecting SDI								
H_1_	ASC → SDI	0.345	8.239	0.000	(0.276, 0.414)		0.193	Accepted
H_2_	PRA → SDI	0.261	5.691	0.000	(0.185, 0.336)		0.078	Accepted
H_3_	ASR → SDI	0.091	2.216	0.013	(0.026, 0.160)	0.684	0.013	Accepted
H_4_	SOC → SDI	0.223	5.657	0.000	(0.160, 0.290)		0.075	Accepted
H_5_	PHB → SDI	0.079	2.256	0.012	(0.021, 0.135)		0.013	Accepted
Factors affecting SDB								
H_6_	SDI → SDB	0.529	14.280	0.000	(0.468, 0.590)	0.280	0.389	Accepted
Mediating effect of SDI(H_7M_)								
H_7a_	ASC → SDI → SDB	0.183	6.452	0.000	(0.138, 0.230)			Mediates
H_7b_	PRA → SDI → SDB	0.138	5.324	0.000	(0.096, 0.182)			Mediates
H_7c_	ASR → SDI → SDB	0.048	2.222	0.013	(0.014, 0.085)			Mediates
H_7d_	SOC → SDI → SDB	0.118	5.386	0.000	(0.083, 0.155)			Mediates
H_7e_	PHB → SDI → SDB	0.042	2.200	0.014	(0.011, 0.073)			Mediates

Source: Author’s data analysis.

*ASC* attitude toward sustainable consumption, *PRA* problem awareness, *ASR* ascription of responsibility, *SOC* sense of community, *PHB* perceived hedonic benefit, *SDI* SHC donation intention, *SDB* SHC donation behavior.

Table 6 also presents the mediating analysis results and indicates that SDI has a positively mediates the relationship between ASC (β = 0.183, p < 0.01), PRA (β = 0.138, p < 0.01), ASR (β = 0.048, p < 0.05), SOC (β = 0.118, p < 0.01), PHB (β = 0.042, p < 0.05) and SDB. The 5% CI and 95% CI values did not straddle zero, thus supporting H_7a_, H_7b_, H_7c_, H_7d_, and H_7e_.

### Structural model

To evaluate the explanatory power of the structural model, that is, data fitness, this study tested the coefficient of determination (R^2^) and the f^2^ effect size recommended by Hair et al.^62^. According to Cohen^66^, an R^2^ value of 0.26 is considered meaningful, 0.13 is fair, and 0.02 is undesirable. In Table 6, the R^2^ for SDI is 0.684, which is above 0.26, and the R^2^ for SDB is 0.280, close to 0.3, which indicates an acceptable level of explanatory power. In addition, Cohen^66^ recommends f^2^ values of 0.02 for small, 0.15 for medium, and 0.35 for large effect sizes. However, in some contexts the observed effect size is smaller than the f^2^ value according to the conventional definition, even when the results produce meaningful effects^67^. In conjunction with the findings of this study, the range of f^2^ between 0.013 and 0.192 consider as a small effect size.

### Multi-group analysis (MGA)

MGA in this study was conducted through the MICOM procedure, which includes three steps: configural invariance, compositional invariance, and equality of a composite’s mean value and variance across groups. As this study ran the MICOM procedure in SmartPLS, configural invariance was automatically established^68^. In Step II, a non-significant permutation value indicated compositional invariance. The results showed that permutation p-values for constructs in gender (male vs. female), frequency of buying clothing (less than or equal to 2 times per month vs. more than 2 times per month), and monthly spending (spending less than or equal to RMB600 vs. spending more than RMB600) were all larger than 0.05, and only 1 out of 7 associations toward education level (Bachelor degree and below vs. Master degree) were lower than 0.05; therefore, the comparison of path coefficients among these four groups were conducted through MGA, and the results are presented in Table 7. Table 7 shows that, for gender, only the path from PHB to SDI had a difference in path coefficient. In the groups with different educational levels, the associations of ASR with SDI, SOC, and SDI were all different. The path coefficients of SDI to SDB also differ based on monthly spending. In addition, no other multi-group differences were found, as all their 2-tailed p-value were greater than 0.05.

**Table 7:** Multi-group analysis.

Associations		Difference	2-tailed p-value	Decision
Gender (male vs. female)				
H1	ASC → SDI	−0.112	0.093	No difference
H2	PRA → SDI	−0.107	0.127	No difference
H3	ASR → SDI	0.041	0.316	No difference
H4	SOC → SDI	0.046	0.296	No difference
H5	PHB → SDI	0.123	0.044	Difference
H6	SDI → SDB	0.005	0.471	No difference
Education level (bachelor degree and below vs. master degree and above)				
H1	ASC → SDI	−0.162	0.040	Difference
H2	PRA → SDI	−0.027	0.394	No difference
H3	ASR → SDI	−0.039	0.339	No difference
H4	SOC → SDI	0.231	0.005	Difference
H5	PHB → SDI	0.009	0.456	No difference
H6	SDI → SDB	0.046	0.314	No difference
Frequency of buying clothing (less than or equal to 2 times per month vs. more than 2 times per month)				
H1	ASC → SDI	−0.015	0.432	No difference
H2	PRA → SDI	0.056	0.282	No difference
H3	ASR → SDI	0.000	0.496	No difference
H4	SOC → SDI	−0.057	0.252	No difference
H5	PHB → SDI	−0.047	0.262	No difference
H6	SDI → SDB	0.091	0.130	No difference
Monthly spending of clothing (spending less than or equal to RMB600 vs. spending more than RMB600)				
H1	ASC → SDI	0.012	0.444	No difference
H2	PRA → SDI	−0.010	0.458	No difference
H3	ASR → SDI	0.022	0.405	No difference
H4	SOC → SDI	−0.001	0.500	No difference
H5	PHB → SDI	−0.049	0.257	No difference
H6	SDI → SDB	0.179	0.012	Difference

Source: Author’s data analysis.

*ASC* attitude toward sustainable consumption, *PRA* problem awareness, *ASR* ascription of responsibility, *SOC* sense of community, *PHB* perceived hedonic benefit, *SDI* SHC donation intention, *SDB* SHC donation behavior.

## Discussion

This study attempted to enhance the understanding of SDI and SDB by considering cognitive (ASC), environmental (PRA and ASR), social (SOC), and personal (PHB) factors. The findings not only confirm the significant influence of constructs related to TIB (cognitive, social, personal) on individuals' intention to donate SHC. Simultaneously, the incorporation of environmental factors (PRA and ASR) into the TIB was undertaken to broaden its scope, while also substantiating the favorable influence of variables PRA and ASR on individuals' inclination to donate secondhand clothing. More specifically, First, the finding towards the relationship between ASC and SDI in this study are consistent with previous studies on sustainable behavior and consider ASC a prerequisite for sustainable intention and behavior^21^. This demonstrates that people with a higher level ASC are more likely to donate SHC in a sustainable manner. Furthermore, the impact of individual ASC on SDI varies depending on educational attainment levels. This observation highlights the significance of residents' educational attainment in influencing their perspectives and behaviors regarding sustainability.

Second, this study showed that environmental factors (PRA and ASR), which were not included in the TIB framework, significantly impacted SDI. Environmental factors have been the primary focus of previous research on sustainable consumption. In addition, PRA and ASR have been confirmed as factors influencing sustainable behavior intention^24, 25^, which is similar to the conclusions of this study. This study demonstrates that PRA has a greater effect on SDI than any other variable except ASC. People’s awareness of environmental issues, as well as the responsibility for both themselves and others, can encourage them to make SHC donations, which will increase their desire to address environmental issues because they will be more willing to live in a sustainable development society.

Third, the findings of this study indicate that SOC, which refers to social factors, is the antecedent of SDI. Similar results have been reported in previous literature regarding pro-environmental behaviors^27, 28, 35^. This interesting finding implies that the intention to choose donations of SHC comes from a person’s sense of belonging to society. In the context of sustainable SHC disposal, SOC is salient in motivating sustainability. A possible explanation is that the ability to be externally constrained by a connection to a communication group may be effective in forming a willingness to engage in beneficial behaviors toward the whole community^35^. Simultaneously, this study also finds that individuals with different educational levels will have different degrees of impact of SOC on SDI. This indicates that the sensitivity of SDI to social attachment could be influenced by personal educational experiences.

Fourth regarding personal factors in the construct model (PHB), the results indicate that positive emotions play a significant role in determining whether people are interested in SHC donations. This result is also supported by prior research on sustainable consumption, namely, that hedonic emotions can serve as an incentive for individuals to engage in sustainable behaviors^39^. This may be owing to the fact that people’s material lives become progressively more abundant, and consequently, they become more interested in spiritual pleasure and more willing to engage in SHC donations that can bring them happiness. This may be one reason TIB, a theory emphasizing personal factors, has become popular in contemporary society. This study also found that gender differences resulted in different effects of PHBs on the intention to donate to SHC. These differences have also been noted in pro-environmental studies^69^, and may be attributed to inherent personality differences between men and women.

Fifth, this study examines the influence of SDI on SDB. According to the findings, there is a statistically significant relationship between the two constructs. As a pre-variable of actual behavior in a vast number of pro-environmental behavior studies, the intention has always been a powerful motivation for the conduct or adoption of actual behavior^43, 44^. This was confirmed in the present study. With the rapid development of the Internet, the sustainable disposal of SHC is an emerging phenomenon. Among the various options SHC disposal methods (discarding, incinerating, donating, swapping and selling etc.), with a high willingness for donation is more conducive to people donating SHC. This study also found that individuals who spent different costs per month on clothing differed in the extent to which their donation intentions translated into actual donation behavior.

Finally, the mediating effect of SDI on the relationship between ASC, PRA, ASR, SOC, PHB, and SDB was examined. This indicates that SDI is partially responsible for the relationship between these predictors and SDB in emerging economies such as China. Previous studies have also confirmed that when the intention is enhanced by cognitive, environmental, social, and personal factors, it becomes an important consideration in pro-environmental behavior^21, 70^. It is evident that the SDI plays a crucial role in constructing the transformation between psychologically related factors and pro-environmental behaviors, particularly SHC donation behavior.

## Implications

### Theoretical implications

Based on a literature review, this study notes that existing research has paid less attention to the adoption of sustainable SHC disposal, and the sustainable development of the clothing industry urgently requires more in-depth research in most countries with a large population base. Therefore, this study makes a valuable contribution regarding the factors that influence sustainable SDB. Additionally, with the theoretical foundation of TIB, this study extends it through including environmental factors at the psychological level. Environmental factors have not received much attention in previous studies that have used the TIB model in a pro-environmental context. However, in studies on sustainable consumption, environmental factors have been proven to have a strong explanatory effect on intention^71^. Through a review of the relevant literature, some important variables that contribute to environmental research were adopted. Therefore, this study adds environmental factors into TIB which can advance the development of the TIB model within the realm of environmental conservation, address certain deficiencies of current research. Furthermore, the utilization of MGA with respect to demographic characteristics sheds light on the significant difference among several causal pathways influenced by the different group which included gender, education, and monthly expenditure on clothing. This not only underscores the significance of these demographic characteristics in understanding sustainable clothing disposal practices but also offers valuable insights for potential avenues of future research in this domain.

Based on a literature review, this study notes that existing research has paid less attention to the adoption of sustainable SHC disposal, and the sustainable development of the clothing industry urgently requires more in-depth research in most countries with a large population base. Therefore, this study makes a valuable contribution regarding the factors that influence sustainable SDB. This study also employs TIB to develop a theoretical framework. Psychologically, cognitive (ASC), environmental (PRA and ASR), social (SOC), and personal (PHB) factors explain the formation of SDI, which ultimately leads to SDB. Among these, environmental factors have not received much attention in previous studies that have used the TIB model in a pro-environmental context. However, in studies on sustainable consumption, environmental factors have been proven to have a strong explanatory effect on intention^71^. Through a review of the relevant literature, some important variables that contribute to environmental research were adopted. Therefore, this study can advance the development of the TIB model within the realm of environmental conservation, address certain deficiencies of current research. Furthermore, the utilization of MGA with respect to demographic characteristics sheds light on the significant difference among several causal pathways influenced by the different group which included gender, education, and monthly expenditure on clothing. This not only underscores the significance of these demographic characteristics in understanding sustainable clothing disposal practices but also offers valuable insights for potential avenues of future research in this domain.

### Practical implications

This study also provides a practical and empirical understanding of the key factors that must be considered when political or commercial parties want to promote donations of SHC. First, consider the importance of cognitive factors, people’s ASC of SHC should receive more consideration. With the rapid growth of the Internet, the rate of global information dissemination has exponentially accelerated. Therefore, online platforms can serve as potent tools for promoting environmentally responsible clothing disposal. Relevant organizations can use emerging social media to establish multichannel, diverse, and multimedia public relations strategies to increase consumers' positive perceptions of ASC, and different implementation plans should be developed for residents with various educational backgrounds.

Second, environmental considerations cannot be disregarded when encouraging residents to donate SHC. To improve the PRA and ASR of residents, it is crucial to foster their understanding of the anthropogenic implications of environmental issues and their underlying factors to increase their environmental awareness and sense of responsibility. The dissemination of knowledge to the public regarding environmental protection is often regarded as a viable strategy for cultivating a collective awareness and concern for the preservation of the environment. Furthermore, it is advisable for both governmental entities and businesses to contemplate the adoption of incentive policies and pertinent regulations aimed at fostering a heightened engagement among residents in the SHC donating practices. This approach would effectively contribute to the establishment of a virtuous cycle, which will offer sustained external motivation for engaging in environmentally protective behaviors.

Third, social factors are among the most influential factors affecting sustainable behavior change. This study confirms that a clear understanding of an individual’s scope of responsibility can motivate them to dispose of SHC sustainably. And the importance of education background although be emphases through the findings from MGA analysis. Education is an effective way for individuals to develop a sense of responsibility, and education on responsible disposal of used clothing should be widely disseminated. The government should strengthen publicity and education on topics related to the sustainable disposal of SHC through online platforms or live lectures that are more accessible to the public. Simultaneously, an improvement in SOC helps stimulate a higher level of SDI. To improve the community’s internal recognition of SDB, the community must provide positive feedback, such as using the influence of celebrities to launch advocacy activities within the community and commend individual donation behavior, on the individual donations of SHC.

Finally, PHB was confirmed as a motivating factor for people’s intention to donate to SHC sustainably. A ritualized donation process is more likely to bring happiness and fulfillment. Relevant organizations may wish to consider issuing certificates to individuals who donate to SHC and encourage individuals to share them online to further spread sustainable SHC behavior. People’s awareness of these issues should also be considered. Raising consumers’ awareness of the environmental problems caused by the failure to reasonably dispose of SHC is also an effective way to improve SDI. The environmental burden of textile waste must be publicized. Related public or social media accounts should be established to regularly push relevant knowledge and news to the public.

## Conclusions

As the main source of textile waste, the ecological pressure caused by SHCs has become the most direct manifestation of their excessive consumption. The better disposal of SHC has become a profound concern for governments and environmental agencies. This study focuses on the sustainable disposal method of second-hand clothing donation, taking into consideration the context of China as an emerging economy. The SHC donation behavior has been chosen due to its development potential and the relatively limited attention it has received from researchers. The examination of Chinese consumers' SHC donation behavior is instrumental in gaining a comprehensive understanding of the determinants influencing consumers' propensity to donate second-hand clothing. Moreover, this investigation can contribute to fostering the sustainable advancement of China's textile and garment sector to a certain degree. Based on a review of the existing literature, this study introduced environmental factors into the TIB model to construct a framework. Through data analysis, the psychological factors influencing intention toward sustainable disposal were identified. The results show that ASC, PRA, ASR, SOC, and PHB have significant positive effects on consumers’ SDI. Based on these findings, theoretical and practical implications are proposed to provide references for future strategies and research related to the sustainable donation of SHC.

### Limitations

Despite these encouraging findings, this study had some limitations. First, as a cross-sectional study, this study cannot accurately capture the changes in the target group in the real world. Besides, this study employs a self-reporting survey to measure SHC donation behaviours, a research method favoured in the social sciences. But a few academics have questioned the accurate of self-reported survey data, especially in some sensitive area^72, 73^. Considering the potential bias, future scholars can use the behaviour experience of respondents as a measurement of actual behavior to improve the precision of data. Additionally, because of the limited geographical scope of the research, only consumer groups within China were investigated; therefore, it may be difficult to extend the results to other regions or even the world. Simultaneously, although the differences within groups are detected in the MGA analysis, this study does not provide in-depth research and discussion on this, and these groups with differences can be considered in future research. Furthermore, considering that the participants in this study encompass individuals who have not previously engaged in the SHC donation behavior. This study does not elaborate on the reasons and channels of SHC donation among the participants, there is a possibility of overlooking potentially valuable research insights. Hence, it is recommended that forthcoming researchers incorporate these past experiences within the scope of investigation, thereby enhancing the research findings. Finally, considering the current situation of SHC disposal in China, the sustainable disposal methods referred to in this study mainly focus on donation and do not involve other methods. Considering the above limitations, future research can expand the overall scope of the study, introduce other sustainable disposal methods, and conduct in-depth and comprehensive research on the statistical characteristics that may influence the results.

### Supplementary Information

Supplementary Information 1.

Supplementary Information 2.

## Data Availability

The original contributions presented in the study are included in the article/Supplementary Material, further inquiries can be directed to the corresponding author/s.
