# Supplementary material for: Modeling the intention and donation of second-hand clothing in the context of an emerging economy
Source: Sci Rep. 2023 Sep 13;13:15106. doi: 10.1038/s41598-023-42437-y (PMC10499903; doi:10.1038/s41598-023-42437-y)
Supplement: Supplementary file 1 — Supplementary Information 1. [file 41598_2023_42437_MOESM1_ESM.docx]

**Supplementary Material - S1.** Survey Instrument

| Code | Items | Source |
| --- | --- | --- |
| ASR1 | I believe we share responsibility for reducing the waste produced by second-hand clothing. | Joshi et al. (2021); Smith et al. (1994) |
| ASR2 | I feel jointly responsible for the negative consequences of second-hand clothing waste. |  |
| ASR3 | I feel jointly responsible for the ecological damage caused by my failure to reduce second-hand clothing waste. |  |
| ASR4 | We are jointly responsible for the environmental deterioration caused by our failure to reduce second-hand clothing waste. |  |
| ASR5 | We are jointly responsible for making environmentally sustainable consumption choices. |  |
| PRA1 | In my opinion, reducing the waste generated by second-hand clothing is a significant approach to lowering pollution. | Khan et al. (2019) |
| PRA 2 | I believe that second-hand clothing waste reduction creates a better environment for future generations. |  |
| PRA 3 | I believe that second-hand clothing waste reduction is a major way to reduce wasteful landfills. |  |
| PRA 4 | I believe that reducing waste from second-hand clothing is a significant way to protect the environment. |  |
| PRA 5 | I think second-hand clothing waste reduction saves money and resources. |  |
| ASR1 | I believe we share responsibility for reducing the waste produced by second-hand clothing. | Zhang et al. (2013);  Wang et al. (2019) |
| ASR2 | I feel jointly responsible for the negative consequences of second-hand clothing waste. |  |
| ASR3 | I feel jointly responsible for the ecological damage caused by my failure to reduce second-hand clothing waste. |  |
| ASR4 | We are jointly responsible for the environmental deterioration caused by our failure to reduce second-hand clothing waste. |  |
| ASR5 | We are jointly responsible for making environmentally sustainable consumption choices. |  |
| SOC1 | I interact with other members of the community regarding sustainable consumption of second-hand clothing (e.g., sell, swap, or donate). | Li-Chun Hsu, (2018); Huang et al., (2020) |
| SOC2 | I share positive feelings with other community members who promote sustainable consumption of second-hand clothing (e.g., sell, swap, or donate). |  |
| SOC3 | I become an important person to other members of the community through my promotion of sustainable consumption of second-hand clothing (e.g., sell, swap, or donate). |  |
| SOC4 | Because of my efforts to promote sustainable consumption of second-hand clothing (e.g., by selling, swapping, or donating), other members of the community respect me. |  |
| SOC5 | People in my community promote sustainable consumption of second-hand clothing (e.g., sell, swap, or donate). |  |
| PHB1 | I feel like I'm helping to solve environmental problems when I sustainably consume second-hand clothing (e.g., sell, swap, or donate). | Hsu and Lin, (2020);  Wiedmann et al. (2009) |
| PHB2 | Sustainable consumption of second-hand clothing (e.g., sell, swap, or donate) makes me feel good. |  |
| PHB3 | I enjoy responsibly consuming second-hand clothing (e.g., selling, swapping, or donating). |  |
| PHB4 | Sustainable consumption of second-hand clothing (e.g., sell, swap, or donate) provides deeper meaning in my life. |  |
| PHB5 | My life is better because I sustainably consume second-hand clothing (e.g., sell, swap, or donate). |  |
|  | | Kim et al. (2021);  Putrevu and Lord (1994) |
| SDI1 | All things considered, I expect to donate my second-hand clothing to people who need it. |  |
| SDI 2 | I can see myself engaging in donating second-hand clothing in the future. |  |
| SDI 3 | I can see myself donating all of my second-hand clothing if possible. |  |
| SDI 4 | It is likely that I will frequently donate second-hand clothing in the future. |  |
| SDI 5 | It is very likely that I will donate second-hand clothing in the future. |  |
| SDB1 | I can reduce second-hand clothing waste by donating second-hand clothing. | Khan et al. (2019) |
| SDB2 | I donate second-hand clothing to reduce the waste of resources. |  |
| SDB3 | I donate second-hand clothing to help others who need it. |  |
| SDB4 | I donated second-hand clothing to prevent these wasteful consumption practices. |  |
| SDB5 | I donate second-hand clothing to reduce environmental degradation. |  |

**Note:** ASC: Attitude toward sustainable consumption; PRA: problem awareness; ASR: ascription of responsibility; SOC: sense of community; PHB: perceived hedonic benefit; SDI: SHC donation intention; SDB: SHC donation behavior.

**Source:** Author’s data analysis
